# Natural History and Conservative Treatment Options in Chiari Malformation Type I in Adults: A Literature Update

**DOI:** 10.7759/cureus.12050

**Published:** 2020-12-13

**Authors:** Fernando Luiz R Dantas, François Dantas, Antônio Carlos Caires, Ricardo V Botelho

**Affiliations:** 1 Neurological Surgery, Biocor Instituto, Belo Horizonte, BRA; 2 Neurological Surgery, Hospital Vila da Serra, Belo Horizonte, BRA; 3 Neurological Surgery, Instituto de Assistência Médica ao Servidor Público Estadual (IAMSPE), São Paulo, BRA

**Keywords:** chiari i malformation, natural history, conservative, treatment

## Abstract

Over the years, knowledge regarding the natural history of Chiari malformation type I (CM-I) has improved. However, there are still uncertainties in the literature regarding asymptomatic and oligosymptomatic patients with CM-I. We performed a literature review in order to determine the natural history of CM-I in symptomatic patients who were not operated and in asymptomatic adult patients. Among symptomatic patients, the most frequent symptom was headache (77%). Cough-related headache alone (34%), migraine-related headache alone (21.4%), and both cough- and migraine-related headaches (21.8%) were the most prevalent forms of headache. Some symptoms improved more effectively with surgical treatment options; cough-related headaches improved by 95% following surgery and by 40% following conservative treatment. Other headache types improved by 93% and 61.5% following conservative and surgical treatments, respectively. Patients diagnosed with asymptomatic or oligosymptomatic CM-I may improve over time or stabilize with conservative treatment. There is great heterogeneity in the literature regarding the diagnostic criteria for CM-I. Owing to the heterogeneity among the articles identified for this review, there is no consensus regarding the precise indications for surgery in asymptomatic patients. The natural CM-I history in asymptomatic patients reveals a favorable evolution.

## Introduction and background

Initially, Chiari malformation type I (CM-I) was described as a group of cerebellar changes that develop owing due to hydrocephalus and that lead to an elongation of the cerebellar tonsils and medial portions of the lower lobes of the cerebellum towards the brainstem [1]. Later, Chiari himself suggested that in addition to hydrocephalus, there was another possible mechanism that could explain the cerebellar alterations, which he considered to be a potential failure in bone development [2,3]. In 1932, the first report on the surgical treatment of this condition was introduced, which described a resection of the cerebellar tissue and bone that was performed on the posterior surface of the malformation through an opening in the dura mater in order to rectify the patient’s hydrocephalus by normalizing the flow of cerebrospinal fluid (CSF) [3]. Since then, foramen magnum decompression has been the most frequently employed surgical technique for the treatment of CM-I in symptomatic patients.

The estimated prevalence of CM-I is one patient in every 1,000 to 5,000 individuals, with the majority of cases occurring sporadically. Recessive or dominant autosomal family inheritance patterns associated with CM-I with incomplete penetrance have also been identified [4]. Through magnetic resonance imaging (MRI) examinations, the prevalence rate of diagnosed CM-I was found to range from 0.56% to 0.77%, with 0.62% of the occurrences observed during necropsies [3,5]. The assessed prevalence rate of the CM-I-syringomyelia complex is 0.24% [6]. In a series comprising 22,591 patients who underwent brain and cervical spine MRI examinations, radiological findings indicating herniations of the cerebellar tonsils extending for a distance greater than 5 mm through the foramen magnum were visualized in 175 patients (0.77%); among these 175 patients, 25 (14%) were clinically asymptomatic. Concerning the asymptomatic patients, syringomyelia was found in only one patient. The associated presence of syringomyelia was uncommon in most of these patients (4.8%) [7]. Tonsillar ectopia was found in approximately 1% of the patients who underwent MRI scans. Furthermore, the association between CM-I and the following skeletal anomalies of the craniocervical junction has been described: odontoid retroflexion (26%) and basilar invagination (12%) [8].

However, the natural history and conservative treatment of symptomatic and asymptomatic patients who did not undergo surgery have seldom been discussed in the literature, and, consequently, we have no clear evidence regarding the appropriate treatment guidelines. Therefore, in this study, we conducted a literature review of the natural history and conservative treatment of adult patients with CM-I, with the aim that our data could be used to assist surgeons in making decisions regarding the best approach that can be employed to treat these patients.

## Review

Methods

We performed an extensive literature search on PubMed (https://www.ncbi.nlm.nih.gov/pubmed/clinical). Literature databases were searched to identify the natural history and conservative treatment in asymptomatic adults with CM-I using the following terms: “Chiari malformation type 1”, AND/OR “Chiari malformation type I”, “conservative”, “asymptomatic”, “natural history”, “non-surgical”, and “non-operative”. The full text of the articles eligible for our study was reviewed and included in the review. We included all the articles related to CM-I, and excluded articles related only to the pediatric population or to congenital syndromes. Articles that involved both a pediatric and adult population were included. We chose MEDLINE as the only assessed database as it is available for a worldwide audience and includes most relevant research.

Radiological diagnosis

Chiari malformation (CM) is a mesodermic malformation characterized by disproportion between the content and capacity of the posterior fossa, leading to overcrowding of neural structures at the level of the foramen magnum [9]. Some authors consider the size of the posterior fossa as a diagnostic criterion for CM-I. The morphometric study of the posterior fossa and craniovertebral junction has been used by the authors to classify CM-I. Patients with CM have an underdeveloped occipital bone; therefore, the posterior cranial fossa becomes shallow. This results in the brain stem and cerebellum sagging into the spinal canal, that is, the pathogenesis of the CM is the insufficiency of the para-axial mesoderm, which is the origin of the occipital bone [10].

There is immense heterogeneity in the literature regarding the criteria that can be used for the diagnosis of CM-I [11]. In this review, we found that some authors define CM-I as a condition in which the herniation of the cerebellar tonsils through the foramen magnum is equal to or greater than 5 mm [12-19]. Ramón et al. defined it as a displacement of the cerebellar tonsils to a distance of 3-5 mm below the foramen magnum [20]. Schuster et al. referred to it an ectopy of the tonsils located below the foramen magnum [21]. We did not find an exact definition in some of the articles [22-26]. Mikulis et al. published an interesting study in which they correlated the position of the cerebellar tonsils with patient age, taking into consideration the following values deemed abnormal for each age group: first decade of life, 6 mm; between 10 and 30 years, 5 mm; between 30 and 79 years, 4 mm; and 3 mm following this age range [27]. McClugage and Oakes, in a recent review on CM-I, used the definition outlining that CM-I is a herniation of the cerebellar tonsils that extends 3 mm below the foramen magnum in children and 5 mm below it in adults [28]. These findings were found in approximately 0.9% of the adult population and 0.6% of the pediatric population [29,30].

**Figure 1:**
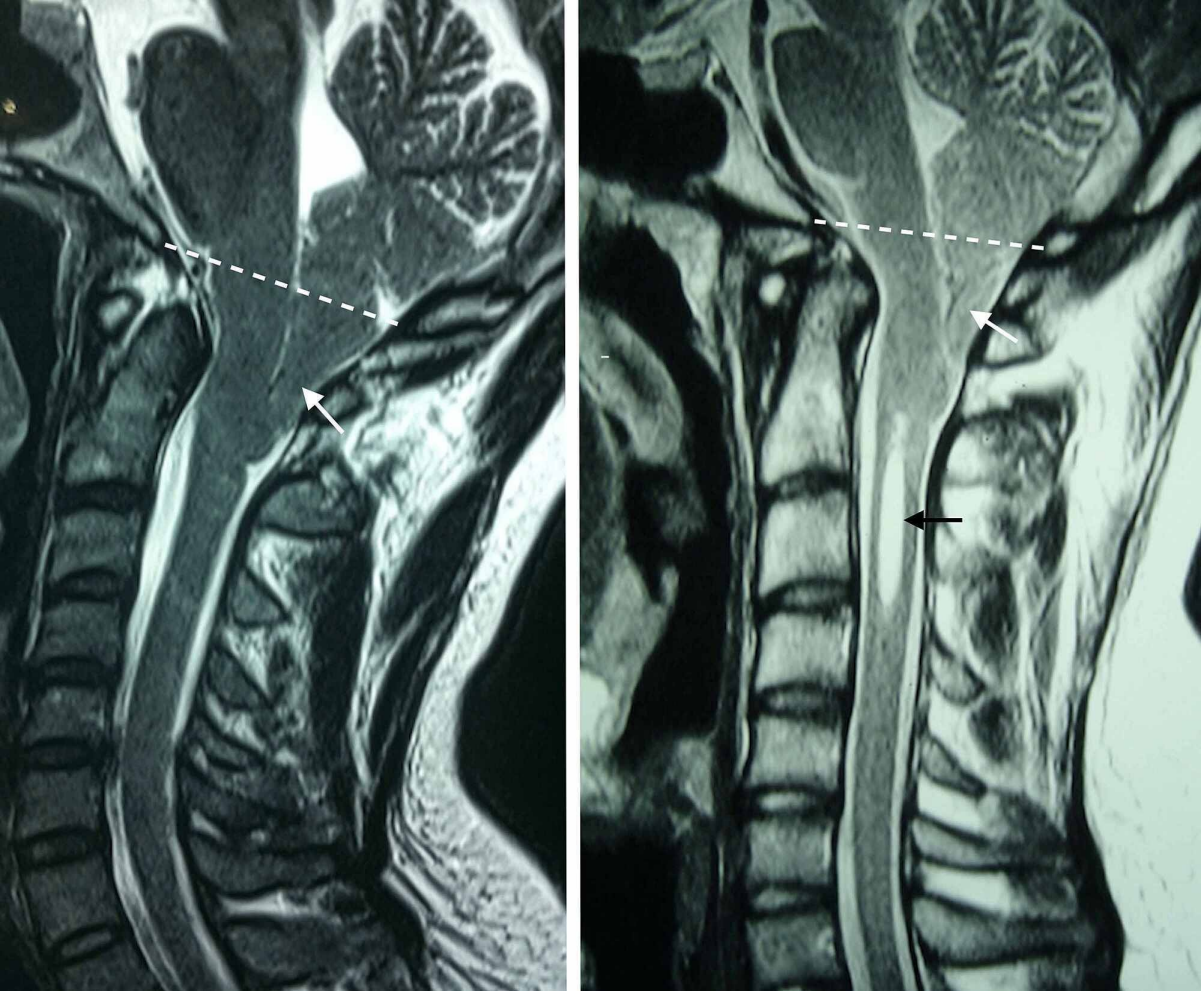
Radiological aspects of CM-I. Herniation of the cerebellar tonsils through the foramen magnum without syringomyelia (left) and associated with syringomyelia (right). The limits of the foramen magnum are represented by the McRae line (white dashed lines), which connects the basion (anterior rim of the foramen magnum) to the opisthion (posterior rim of the foramen magnum). Cerebellar tonsils are indicated by the white arrows, and syringomyelia is indicated by the black arrow. CM-I, Chiari malformation type I

Radiological aspects of CM-I are shown in Figure 1.

Clinical diagnosis

The clinical symptoms associated with CM-I are a result of the following three categories of possibilities: symptoms related to CSF obstruction, those related to the compression of the craniocervical junction (brainstem, cerebellum, or cranial nerves), or those related to spinal dysfunction and syringomyelia [28]. The most commonly observed symptom is an occipital headache that worsens with coughs or neck extensions and actions involving the Valsalva maneuver, such as coughing or exertion; additionally, some patients report experiencing visual disturbances, paresthesia, weakness in the limbs, dysphagia, or an altered vomiting reflex [8].

In the two major series included in this research [15,16], the most frequently reported symptoms were headaches of any type (77%), cough-related headache only (34%), migraine-related headache only (21.4%), and both cough- and migraine-related headaches (21.8%). The other observed symptoms were paresthesia (34%), cerebellar symptoms (27%), dysphagia (21%), dysphagia or apnea (9%), and nausea (13%). Drop attacks (4%) were rarely observed.

Some patients with asymptomatic CM-I develop symptoms after undergoing trauma, and there are reports on deaths occurring during sports [31-33]. Neck and left shoulder pain accompanied by paresthesia in the upper limbs was described in the case reported by Sung et al. [22].

Additional rare symptoms, such as acute quadriparesis [14], trigeminal neuralgia [20], cerebellar symptoms following intraoperative lumbar drainage [34], and sleep apnea [17], can also be observed in some patients with CM-I. Despite the rare presentation of sleep apnea, studies that involved polysomnography examinations revealed abnormalities in approximately 59-70% of patients with CM-I [35,36].

Massimi et al. described three cases (an adult aged 38 years and two children aged 1 and 2.5 years) involving the acute onset of symptoms. The adult patient presented with acute respiratory failure five days following an intestinal occlusion surgical procedure, and a cerebral MRI examination revealed hydrocephalus; furthermore, in his adolescence, he was diagnosed with Noonan syndrome accompanied by CM-I and syringomyelia resulting from a thoracic deformity and scoliosis [25].

Natural history and surgical indications

Due to the heterogeneity among the articles identified for this review, there is still no consensus regarding the precise indications for surgery in the case of asymptomatic patients with CM-I. Some of the articles that were identified for inclusion in this review and that outlined the criteria for surgical indications are summarized in Table 1.

**Table 1:** Criteria for surgical intervention in CM-I NA, not applicable; CSF, cerebrospinal fluid; CM-I, Chiari malformation type I

Author, year	Article type/number of patients	Reason for surgical indication
Massimi et al., 2011 [25]	Case series/3	Clinical or neurophysiological deterioration
Ramón et al., 2011 [20]	Review/NA	Progressive posterior fossa or spinal cord symptoms/signs; hydrocephalus or syringomyelia
Kalb et al., 2012 [12]	Retrospective/104	Symptomatic patients, with or without syringomyelia
Schneider et al., 2013 [14]	Case report/1	Neurological worsening
Chavez et al., 2014 [15]	Cohort/177	Presence of cough-associated headaches; effect on quality of life; large or growing syringomyelia; abnormal neurological examination results or myelopathy; abnormalities in the descending tonsillar CSF flow
Killeen et al., 2015 [16]	Retrospective cohort/68	Patients with radiological examination findings and clinical symptoms associated with CM-I; cough headaches
Leu, 2015 [17]	Review/NA	Severe sleep-related breathing disorders
McClugage and Oakes, 2019 [28]	Review/NA	Patients with classic Valsalva-induced headaches; patients with an associated syrinx; patients with neurological sequelae associated with pathologies of the foramen magnum and cervicomedullary junction; or patients with a lower cranial nerve dysfunction
Atchley et al., 2020 [19]	Review/NA	Cough-related headaches; symptomatic syringomyelia; hydrocephalus; brainstem compression
Frič and Eide, 2020 [26]	Review/NA	CSF flow obstruction

Regarding symptomatic patients, some of the symptoms improve more effectively following surgical treatment options compared to following conservative ones. Chavez et al. obtained an improvement of 94.6% in cough-related headaches following surgery, whereas they obtained a 40% improvement in this symptom following conservative treatment. The other types of headaches improved by 92.9% with surgery and by 61.5% with conservative treatment [15].

Killeen et al. obtained less favorable results associated with improving cough-related headaches following surgery. In 37% of the cases, there was an improvement; however, in approximately 52% of the cases, there were no changes, and in 11% of the cases, a worsening of the symptoms was observed [16]. In this same study, the symptoms of ataxia and paresthesia improved more effectively in patients who underwent surgeries compared to those who received conservative treatment.

Among the included articles, a degree of variability in the surgical indications was demonstrated. Ramón et al. indicated surgery to treat cough-related headaches in only 25% of their patients [20].

In a series including 104 patients (17 were pediatric), Kalb et al. demonstrated better outcomes following surgery in patients without syringomyelia and in those with mild symptoms compared to patients with syringomyelia (73% versus 62%). The improvement in sensory disorders was more evident in the group without syringomyelia compared to patients with syringomyelia (78% versus 56%) [12].

In the literature, the treatment of syringomyelia for patients with asymptomatic CM-I is very controversial, but, currently, there is a trend in favor of administering conservative treatment options accompanied with clinical-radiological monitoring [12]. In this review, we found that the presence of syringomyelia was used as an indicator for surgery by some of the authors [23], whereas it was not taken into account in other series [22].

Sung et al. demonstrated a spontaneous resolution of syringomyelia in a 43-year-old patient with neck pain and numbness in the upper limbs. An MRI examination revealed a small C2-T2 syringomyelic cavity and a wide T2-T10 syringomyelia with a maximum diameter of 9 mm. A year later, a control MRI scan revealed a significant reduction in the size of the cavity; however, the patient's symptoms persisted [22].

Miele et al. reported on a case involving the spontaneous resolution of syringomyelia and CM-I (14 mm herniation of the tonsils) in a 36-year-old patient who was being followed-up due to minimal Chiari symptoms. She underwent cranial surgery five years after the follow-up period to treat a bleeding frontal cavernoma. A control MRI scan performed two years after the surgical procedure revealed that her syringomyelia was almost completely resolved and that the cerebellar tonsils were 4 mm below the foramen magnum [13].

Nishizawa et al. analyzed nine patients with syringomyelia that was incidentally identified via imaging examinations during a mean follow-up period of 11.2 ± 0.7 years and compared the results with those of a group of 11 symptomatic CM-I patients. There were no differences found between the images of the symptomatic and asymptomatic groups. Only one patient in the asymptomatic group required surgery seven years following the first visit. Parameters, such as the axial diameter and longitudinal extension of a syrinx and extension of the herniation of the tonsils, had no prognostic value. The authors concluded that in patients with asymptomatic CM-I, syringomyelia resulted in benign lesions [37].

In a systematic review, Schuster et al. demonstrated that the recurrence rate of residual postoperative syringomyelia ranged from 0% to 22% (mean: 6.7%). Furthermore, they directed attention to arachnoid adhesions present at the level of the cerebellar tonsils as a factor that contributes to the persistence of changes in the CSF flow. In cases involving adequate decompression in patients with large syringomyelic cavities and in those with residual syringomyelia, it was uncovered that a new approach may not lead to clinical improvement in addition to it resulting in additional risks for the patient [21].

Gaunt et al. reported on a case involving the spontaneous regression of CM-I in a 58-year-old patient who began to experience paresthesia in the upper limbs and trigeminal neuralgia and who had cerebellar tonsils extending 25 mm below the foramen magnum. On performing a new control MRI scan two years later, the patient exhibited normal tonsil positions and partial improvement of neuralgia owing to drug control and without any treatment performed [18].

Langridge et al. believe that the decision to operate on symptomatic patients with severe headaches, ataxia, or paresthesia is subjective and depends on a balance among the severity of symptoms, effect on the quality of life, and potential postoperative complications. In asymptomatic patients, there is little evidence in the literature to suggest the requirement for surgery based only on radiological findings. Patients with moderate symptoms should be observed, as the symptoms may remain unchanged or improve over time, with acute deterioration being rare [11].

In a recent review on the topic, McClugage and Oakes recommend the administration of surgical treatment to only those patients with symptoms directly related to Chiari or syringomyelia, and they propose that surgery is indicated in the following conditions: patients experiencing classic headaches induced by the Valsalva maneuver, patients with associated syringomyelia, and patients with neurological conditions associated with pathologies of the foramen magnum and cervical medullary junction or a dysfunction of the cranial nerves [28].

In the review by Atchley et al., no definitive or consistently reliable imaging predictors were identified to prospectively decide which patients could benefit the most from a surgical intervention. The presence of preoperative syringomyelia was associated with a scoliosis curve of >20º, tonsil position of 13 ± 5.3 mm, and clivus gradient of 55.2º ± 7.4º. There was no correlation between postoperative improvement and the presence or absence of basilar invagination. Following posterior fossa decompression, a scoliosis curve of <20º and small syrinx were associated with outcomes indicating stability or improvement. Although syringomyelia morphology and length were associated with an improvement in syringomyelia size following posterior fossa decompression, these same characteristics were not associated with symptomatic results. The authors found eight studies that evaluated the presence of syringomyelia as a predictor of postoperative outcomes and found variable results. In six studies, no statistically significant results were found related to the association between the presence of syringomyelia in the preoperative period and an improvement of symptoms following the decompression of the posterior fossa. This review illustrates the discrepancies among the available studies and the heterogeneity among the imaging findings in the CM-I population. In addition, this study highlights the lack of high-quality evidence associated with outcome predictors in CM-I. Therefore, the authors conclude that the treatment option for each patient should be considered based on their clinical histories and physical examination results [19].

Other imaging examination modalities have been used to evaluate patients with CM-I and to assess the results in relation to surgical indications. Krueger et al. studied and compared the CSF flow rate in the posterior fossa using MRI in symptomatic and asymptomatic CM-I patients (26 versus 24 patients). There was no difference in the peak CSF velocity in the foramen magnum between the two groups. During the examination, the position of the head, including the neutral, flexion, or extension positions, did not affect the velocity of the CSF in both groups. Therefore, they concluded that studying the CSF flow velocity in the foramen magnum does not serve to differentiate symptomatic from asymptomatic patients. The value of the results of these studies in determining a need for surgical intervention is still speculative [24].

Sporting activities and CM-I

CM-I has also been a controversial topic in asymptomatic patients who practice sporting activities [31-33]. Harrell and Barootes reported on a case involving a 19-year-old high-performance athlete, who underwent an MRI examination due to frontal headaches and a normal neurological examination. The cerebellar tonsils were located 8-9 mm below the foramen magnum. Sinusitis was diagnosed, and treatment was administered. During a follow-up period of four years, the patient responded well, remained asymptomatic, and was instructed to resume his sporting activities; furthermore, he continued on to play professional football. However, the authors propose a protocol that involves the removal of an athlete with CM-I from sports practice in some situations: the presence of syringomyelia, obliteration of the subarachnoid space, evidence of the indentation of the anterior spinal cord, and presence of symptoms that can be explained by the obstruction of the CSF flow [23].

Spencer and Leach performed a systematic review in this regard and found 21 cases involving patients with CM-I that deteriorated following trauma. The authors noted that the risk of acute deterioration or death in these patients is very low, although it is higher in relation to the general population. There was not enough evidence to differentiate these outcomes between adults and children. The authors recommend that there be no restrictions associated with the participation of asymptomatic patients with CM-I and without syringomyelia in contact sports but that the subject must be well discussed with the patients and their families [32].

Limitations of this study

The article is a narrative review and, therefore, does not follow the standard Preferred Reporting Items for Systematic Reviews and Meta-Analyses (PRISMA) guidelines for systematic reviews. The possibility of bias remains both within individual studies and across studies since we could not perform a full quality assessment.

## Conclusions

Examining the natural history of patients with asymptomatic CM-I reveals a favorable evolution. CM-I patients with mild symptoms should be followed up carefully. Patients diagnosed with asymptomatic CM-I or those with mild symptoms may improve over time or stabilize with conservative treatment. While exploring indications to operate on these patients, specialists must consider the risks and benefits of the surgical procedure, taking into account that serious deterioration can occur in rare cases. The decision to operate should be based on the severity and duration of symptoms. For patients with CM-I who are asymptomatic and who have no signs of syringomyelia, no restrictions are required for their participation in contact sports; however, the subject must be well discussed with the patients and their families.
